# Visual data: a new tool to improve the presentation of clinical trial results

**DOI:** 10.31744/einstein_journal/2020AE4729

**Published:** 2019-11-14

**Authors:** Raphael Mendes Ritti-Dias, Marilia de Almeida Correia, Breno Quintella Farah

**Affiliations:** 1 Universidade Nove de Julho São PauloSP Brazil Universidade Nove de Julho, São Paulo, SP, Brazil.; 2 Universidade Federal Rural de Pernambuco RecifePE Brazil Universidade Federal Rural de Pernambuco, Recife, PE, Brazil.

**Keywords:** Randomized controlled trials as topic, Biostatistics, Data display, Non-randomized controlled trials as topic

## Abstract

Randomized controlled trials are known to be the best tool to determine the effects of an intervention; however, most healthcare professionals are not able to adequately understand the results. In this report, concepts, applications, examples, and advantages of using visual data as a complementary tool in the results section of original articles are presented. Visual simplification of data presentation will improve general understanding of clinical research.

## INTRODUCTION

Randomized controlled trials (RCT) have been considered the most powerful experimental design tool to determine the effects of therapeutic interventions on patient outcomes. When performed well, RCT are considered the gold standard design to support decisions in clinical practice.

In order to appropriately interpret RCT results, the readers of a published trial need complete, clear, and transparent information on the trial methods and findings.^(^^1^^)^ Although important initiatives, such as Consolidated Standards of Reporting Trials (CONSORT), have brought significant improvement to RCT reporting, the adequate interpretation of trial results remains a challenge for researchers and practitioners.

An adequate understanding of statistical methods has been considered a major problem in translating RCT results to the scientific community. Although efforts have been made to improve the presentation of RCT results, the literature indicates that the majority of health practitioners are not able to adequately understand the results of clinical research. For example, in a multicenter study, Johnston et al.^(^^2^^)^ observed that less than 30% of physicians had a correct understanding of frequent statistical methods employed in RCT, such as mean difference and minimal important difference. These results become more alarming when considering the variety and complexity of statistical procedures that have been used in RCT.^(^^3^^)^

A previous study identified that most practitioners perceived dichotomic variables as more valuable than continuous data for clinical decision making.^(^^2^^)^ The simplification of presentation can be a way to improve the understanding of RCT results, helping to fill the gap between research and practice.

## VISUAL DATA: DEFINITION AND EXAMPLES

**Figure 1:**
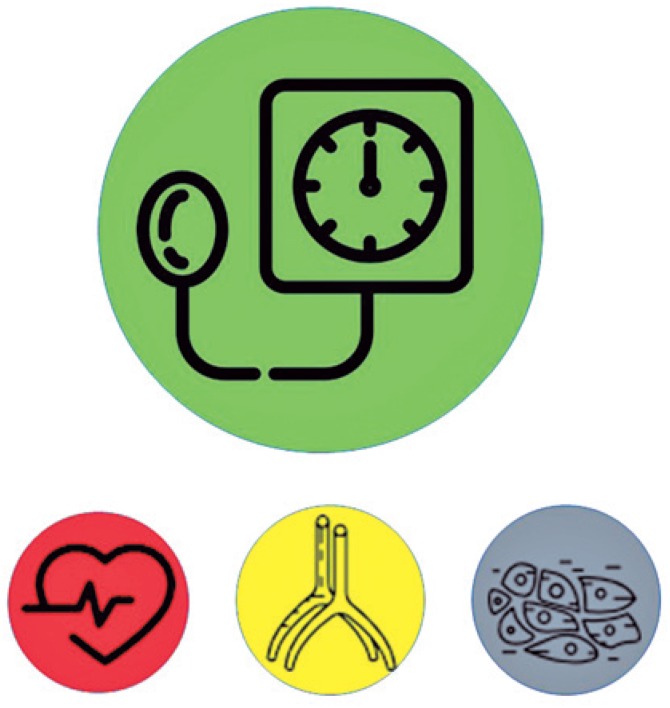
Identification of improvement (green), worsening (red), no change (yellow) or missing data (gray) of an intervention with primary (large symbol) and secondary (smaller symbols) variables

Visual data has been widely used in business to facilitate the identification of problems in process chains. The idea is to provide visual schemes according to specified criteria, thus making the identification of successful and unsuccessful cases clearer. An example of single visual data is presented in figure 1.

In the figure, the large circle represents the main outcome (blood pressure), while the other three small circles represent the secondary outcomes (heart rate, arterial stiffness, and endothelial function). Inside each circle, a visual image with a symbol of each outcome was included to facilitate figure interpretation. The results of the intervention in each variable are indicated by the colors inside each circle (improvement, green; maintenance, yellow; worsening, red; missing data, gray). Thus, in the exemplified case, the subject improved blood pressure, maintained arterial stiffness and worsened heart rate. The endothelial function data was not collected.

**Figure 2:**
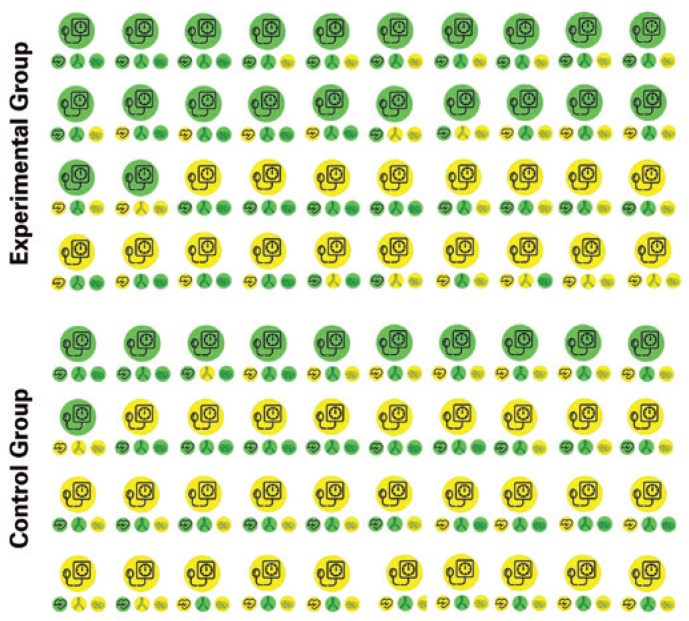
Data from a complete study including 80 subjects, 40 in the Experimental Group and 40 in the Control Group

Figure 2 presents the data of an entire study including 80 subjects, 40 in Experimental Group and 40 in Control Group. Despite the inclusion of several subjects in the same chart, the interpretation of the results is clear. In the figure, it is possible to observe that most subjects in the Experimental Group improved the primary outcome, while only a few subjects in the Control Group improved in this variable. In addition, there was heterogeneity in response for secondary outcomes among subjects of the Experimental Group. For comparison, the same data of figure 2 are presented in table 1, using the presentation pattern commonly used in RCT.

**Table 1:** Data from the figure 2 presented as mean (standard deviation)

Variables	Control Group		Experimental Group		Time effect	Group effect	Interaction effect
	Pre	Post	Pre	Post			
Blood pressure, mmHg	139 (3)	137 (2)	139 (2)	134 (2)	<0.001	<0.001	<0.001
Heart rate variability, ms	24.0 (3.5)	24.9 (4.2)	23.4 (4.0)	24.7 (3.4)	0.069	0.495	0.648
Arterial stiffness, m/s	9.0 (0.6)	7.9 (0.7)	8.9 (0.6)	7.8 (0.6)	<0.001	0.315	0.733
Endothelial function, %	12.2 (1.5)	11.9 (1.5)	12.1 (1.4)	12.0 (1.4)	0.461	0.747	0.573

## ADVANTAGES OF VISUAL DATA

**Figure 3:**
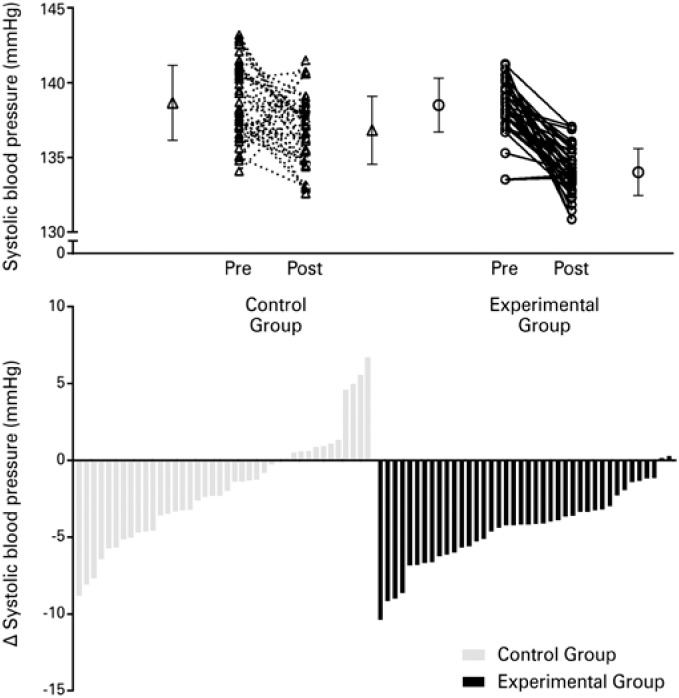
Individual data presented as lines or bars

The presentation of individual data has increased in recent years in order to improve the reporting of RCT. In most cases, data is presented in line or bar charts (Figure 3), being each line or bar a subject. In most cases, only the primary outcome individual data are presented. When secondary outcomes are presented, they are included in different figures. A clear advantage of the proposed visual data compared to these graphs is the integrative view of intervention effects on primary and secondary outcomes for each subject. This allows for a more comprehensive interpretation, helping physicians better understand the effects of interventions on primary and secondary outcomes simultaneously.

**Figure 4:**
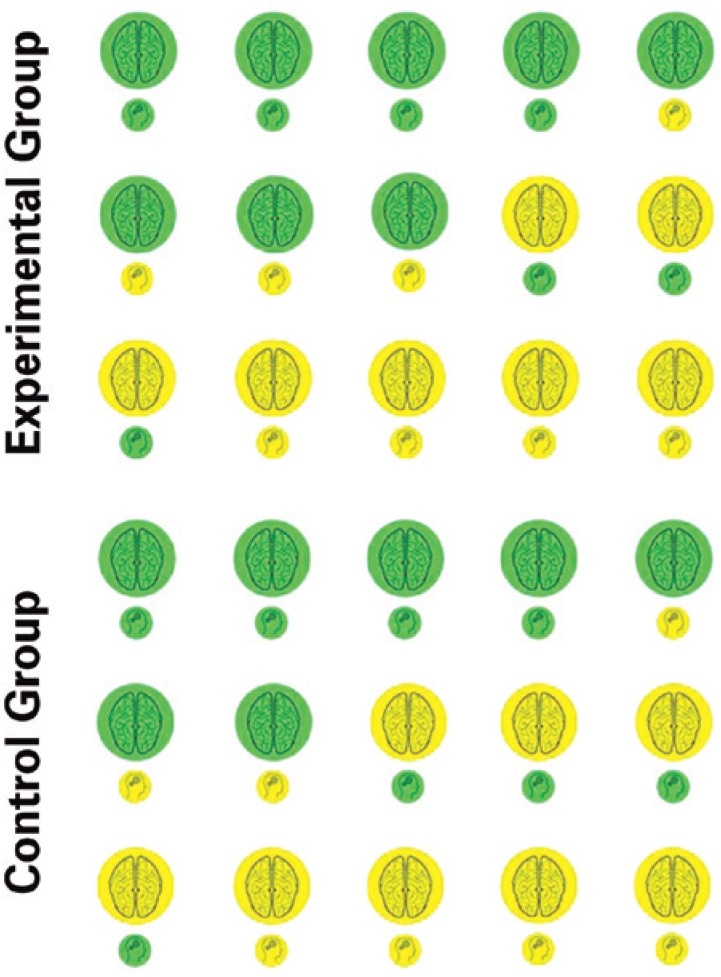
Representation of responses of an intervention in the Experimental Group and Control Group. Green indicates improvement, and yellow, maintenance

The use of visual data implies that continuous variables should be transformed from numerical to categorical variables (improved, maintained or worsened). From a clinical point of view, this clarifies the clinical relevance of results. The use of categorical variables is also better for physicians. A previous study with 531 physicians from 8 countries reported that they best understood the dichotomous presentations of continuous outcomes and perceived them to be the most useful.^(^^2^^)^ Hence, the utilization and extraction of data for healthcare professionals may be enhanced with visual data. A typical case in which this is helpful is presented in table 2 and figure 4. As table 2, statistically significant changes were observed in primary outcomes. However, visual data analysis suggested that no clinically meaningful alteration was verified.

**Table 2:** Data from the figure 4 presented as mean (standard deviation)

Variables	Control Group		Experimental Group		Time effect	Group effect	Interaction effect
	Pre	Post	Pre	Post			
Cerebral blood flow, mL/min	52.0 (0.2)	54.3 (0.2)	52.1 (0.2)	52.2 (0.3)	<0.001	<0.001	<0.001
Cognitive, score	18.8 (0.6)	20.3 (0.7)	19.1 (0.6)	20.2 (0.8)	<0.001	0.488	0.213

**Figure 5:**
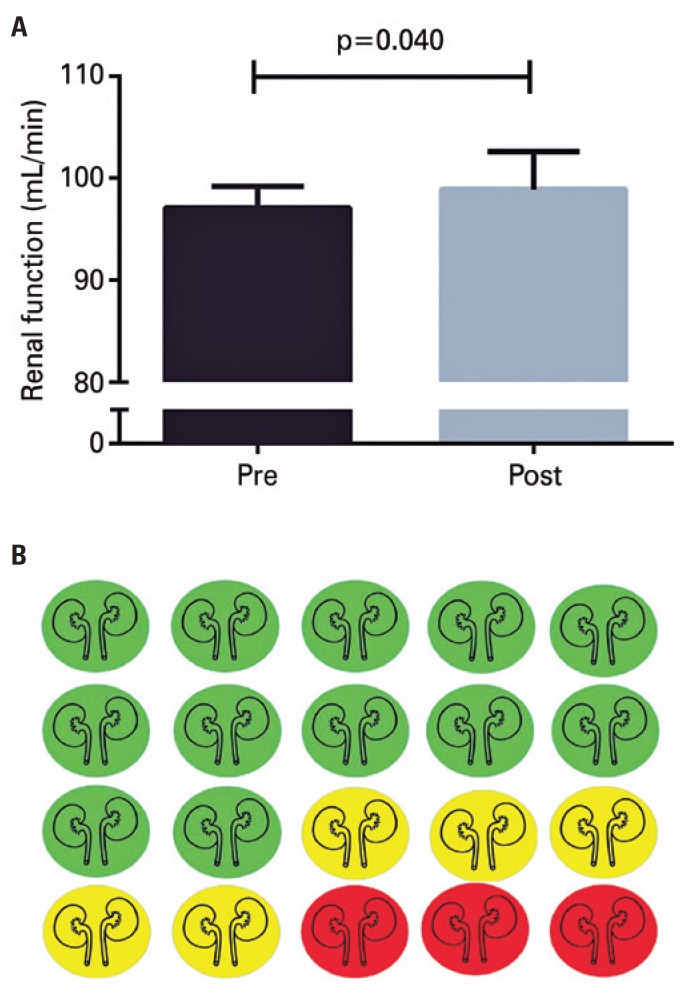
Pre- and post-intervention values for renal function (panel A) and visual data of individual responses. Green indicates improvement, yellow, maintenance, and red, worsening (panel B)

In addition, although the interventions aimed to improve the main outcome, it is possible that it may cause unwanted effects (adverse reactions) in some individuals. Still, the mean comparison, represented in numbers, does not allow identification of unwanted effects after intervention. For example, figure 5 (panel A) shows that, after an intervention, there was a statistically significant improvement in renal function − pre-values: 97.1 (2.1) *versus* post-values: 98.9 (3.7), with p=0.040.

However, 25% (n=5) of the subjects did not show any improvement, and 15% (n=3) presented adverse reactions, as shown in figure 5 (panel B).

The use of visual data may favor the dissemination of RCT results through non-scientific communication, such as television, blog and social networks − media commonly used to inform the non-academic public interested in scientific research.^(^^4^^)^ This is an important topic given the growing interest of scientific journals in disseminating their articles to main public through social media (*e.g*. Facebook, Twitter and Instagram).^(^^5^^)^

In this sense, the current proposal is a first suggestion to use visual data as a tool to improve the interpretation of clinical trial results. Researchers and physicians are invited to employ this tool in different areas, to identify its feasibility in different contexts, allowing to refine and improve the utilization of visual data.

## DEFINING CUT-OFF POINTS

Most physicians prefer dichotomous results to understand RCT results.^(^^2^^)^ It is known that for some outcomes, this type of result may leave doubts about the interpretation of findings.^(^^3^^)^ Cut-off point is defined as a main point in visual data. Several methods have been proposed to define the individual clinically relevant effect of interventions, and depending on the method employed, interpretation of visual data varies drastically.

The following main methods have been used to classify the effects of interventions:

– Delta zero: for some outcomes that usually change with treatment, if the delta (pre- and post-values) differs from zero, it indicates a response.^(^^6^^)^ This method can easily be argued, because it does not consider variations, such as reliability, random variability, and individual variations.
– Changes based on the risk: for some health variables, longitudinal studies have established cut-off points associated with health events. In this sense, some studies have used these clinically relevant values to classify the outcomes in RCT. For example, it is possible to classify subjects as responder if the blood pressure reduces to 3mmHg, which is the value associated with fatal or non-fatal cardiovascular events. This criterion could be used for other variables as arterial stiffness (1m/s),^(^^7^^)^ resting heart rate (75bpm),^(^^8^^)^ flow-mediated dilation (1%),^(^^9^^)^ and biomarkers,^(^^10^^)^ among others.
– Tertiles and quartiles: outcomes have no established cut-off points, and some studies have used arbitrary values based on the median, tertiles, quartiles or percentiles, which can be contested.^(^^11^^,^^12^^)^
– Minimal detectable difference: it defines the difference between the means of a treatment and the control that must exist in order to conclude that there is a significant effect, beyond any measurement error with a given level of confidence, usually at 95% confidence level. For this, one must know the error of the measurement of the variable under analysis.^(^^13^^)^
