# Supplementary material for: Visual data: a new tool to improve the presentation of clinical trial results
Source: Einstein (Sao Paulo). 2019 Nov 14;18:eAE4729. doi: 10.31744/einstein_journal/2020AE4729 (PMC6896661; doi:10.31744/einstein_journal/2020AE4729)
Supplement: Translation

# Translation: Dados visuais: uma nova ferramenta para melhorar a interpretação dos resultados de ensaios clínicos

**Authors:** Raphael Mendes Ritti-Dias, Marilia de Almeida Correia, Breno Quintella Farah

**Affiliations:** 1 São PauloSP Brasil Universidade Nove de Julho, São Paulo, SP, Brasil.; 2 RecifePE Brasil Universidade Federal Rural de Pernambuco, Recife, PE, Brasil.

**Keywords:** Ensaios clínicos controlados aleatórios como assunto, Bioestatística, Apresentação de dados, Ensaios clínicos controlados não aleatórios como assunto

## Abstract

Ensaios clínicos randomizados são conhecidos por serem a melhor ferramenta para determinar os efeitos de uma intervenção. No entanto, a maioria dos profissionais de saúde não é capaz de compreender os resultados de forma adequada. Neste artigo, são apresentados conceitos, aplicações, exemplos e vantagens do uso de dados visuais como uma ferramenta complementar na seção dos resultados de artigos originais. A simplificação da visualização da apresentação dos dados deve melhorar o entendimento geral de pesquisas clínicas.

## INTRODUÇÃO

Os ensaios controlados randomizados (ECR) são considerados a ferramenta de desenho experimental mais poderosa para determinar os efeitos de intervenções terapêuticas nos desfechos de pacientes. Quando bem realizados, são considerados o desenho padrão-ouro para dar suporte à tomada de decisões na prática clínica.

Para interpretar corretamente os resultados dos ECR, os leitores de um ensaio publicado precisam de informações completas, claras e transparentes sobre sua metodologia e seus achados.^(^^1^^)^ Apesar de iniciativas importantes, como a *Consolidated Standards of Reporting Trials* (CONSORT), terem proporcionado melhorias consideráveis à publicação dos ECR, a interpretação correta dos resultados de estudos continua sendo um desafio para os pesquisadores e profissionais de saúde.

Ter uma compreensão apropriada dos métodos estatísticos é um grande problema na tradução dos resultados dos ECR para a comunidade científica. Embora algumas iniciativas já tenham sido feitas para melhorar a apresentação de resultados dos ECR, a literatura indica que a maioria dos profissionais de saúde não consegue compreender corretamente os resultados de pesquisas clínicas. Por exemplo, em um estudo multicêntrico, Johnston et al.^(^^2^^)^ observaram que menos de 30% dos médicos entenderam corretamente os métodos estatísticos empregados com frequência em ECR, como a diferença média e a diferença mínima importante. Esses resultados tornam-se mais alarmantes quando se consideram a variedade e a complexidade dos procedimentos estatísticos usados nos ECR.^(^^3^^)^

Um estudo anterior identificou que a maioria dos profissionais de saúde considera as variáveis dicotômicas mais valiosas que dados contínuos, para fins de tomada de decisões clínicas.^(^^2^^)^ A simplificação da apresentação pode ser uma forma de melhorar a compreensão dos resultados dos ECR, ajudando a preencher a lacuna entre a pesquisa e a prática.

## DADOS VISUAIS: DEFINIÇÃO E EXEMPLOS

Os dados visuais têm sido amplamente usados em negócios para facilitar a identificação de problemas nas cadeias de processos. A ideia é fornecer esquemas visuais, de acordo com critérios especificados, tornando mais clara a identificação de casos de sucesso e insucesso. A figura 1 apresenta um exemplo de dados visuais avulsos.

**Figura 1:** Identificação de melhora (verde), piora (vermelho), não alteração (amarelo) ou dados ausentes (cinza) de uma intervenção com variáveis primárias (símbolo maior) e secundárias (símbolos menores)

Na figura, o círculo grande representa o desfecho principal (pressão arterial), enquanto os outros três círculos pequenos representam os desfechos secundários (frequência cardíaca, rigidez arterial e função endotelial). Dentro de cada círculo, uma imagem visual com um símbolo de cada desfecho foi incluída, para facilitar a interpretação da figura. Os resultados da intervenção em cada variável são indicado pelas cores dentro de cada círculo (melhora, verde; manutenção, amarelo; piora, vermelho; dados ausentes, cinza). Assim, no caso exemplificado, o participante teve melhora da pressão arterial, manteve a rigidez arterial e teve piora da frequência cardíaca. Não foram coletados dados de função endotelial.

A figura 2 apresenta os dados de um estudo que incluiu 80 participantes, 40 no Grupo Experimental e 40 no Grupo Controle. Apesar da inclusão de vários participantes no mesmo gráfico, a interpretação dos resultados é clara. Na figura, é possível observar que a maioria dos participantes do Grupo Experimental teve melhora no desfecho primário, enquanto apenas poucos participantes do Grupo Controle apresentaram melhora nessa variável. Além disso, houve heterogeneidade na resposta para desfechos secundários entre os participantes do Grupo Experimental. Para fins de comparação, os mesmos dados da figura 2 são apresentados na tabela 1, usando o padrão de apresentação frequentemente usado em ECR.

**Figura 2:** Dados de um estudo completo, incluindo 80 sujeitos, 40 no Grupo Experimental e 40 no Grupo Controle

**Tabela 1:** Dados da figura 2 apresentados como média (desvio padrão)

Variáveis	Grupo Controle		Grupo Experimental		Efeito de tempo	Efeito de grupo	Efeito de interação
	Pré	Pós	Pré	Pós			
Pressão arterial, mmHg	139 (3)	137 (2)	139 (2)	134 (2)	<0,001	<0,001	<0,001
Variabilidade da frequência cardíaca, ms	24,0 (3,5)	24,9 (4,2)	23,4 (4,0)	24,7 (3,4)	0,069	0,495	0,648
Rigidez arterial, m/s	9,0 (0,6)	7,9 (0,7)	8,9 (0,6)	7,8 (0,6)	<0,001	0,315	0,733
Função endotelial, %	12,2 (1,5)	11,9 (1,5)	12,1 (1,4)	12,0 (1,4)	0,461	0,747	0,573

## VANTAGENS DOS DADOS VISUAIS

A apresentação de dados individuais aumentou nos últimos anos, para melhorar o reporte de resultados de ECR. Na maioria dos casos, os dados são apresentados em gráficos de linhas ou barras (Figura 3), sendo cada linha ou barra um participante do estudo. Na maioria dos casos, apenas os dados individuais dos desfechos primários são apresentados. Quando os desfechos secundários são apresentados, estes são incluídos nas diferentes figuras. Uma vantagem clara dos dados visuais propostos, em comparação aos gráficos, é a visão integrada dos efeitos das intervenções nos desfechos primários e secundários para cada participante do estudo. Isso permite a interpretação mais abrangente, o que ajuda os clínicos a compreender melhor os efeitos das intervenções nos desfechos primários e secundários simultaneamente.

**Figura 3:** Dados individuais apresentados em linhas ou barras

O uso de dados visuais implica que as variáveis contínuas sejam transformadas de variáveis numéricas a categóricas (melhora, manutenção ou piora). Do ponto de vista clínico, isso esclarece a relevância clínica dos resultados. O uso de variáveis categóricas também é melhor para os médicos. Um estudo anterior, com 531 médicos de 8 países, relatou que os médicos entendiam melhor as apresentações dicotômicas de desfechos contínuos, e as achavam mais úteis.^(^^2^^)^ Desse modo, o uso e a extração de dados para profissionais de saúde podem ser melhorados com dados visuais. Um caso típico em que isso é útil é apresentado na tabela 2 e na figura 4. Conforme a tabela 2, foram observadas alterações estatisticamente significativas nos desfechos primários. No entanto, a análise dos dados visuais sugere que não foi verificada alteração clinicamente significativa.

**Figura 4:** Representação das respostas de uma intervenção no Grupo Experimental e Grupo Controle. Verde indica melhora e amarelo manutenção

**Tabela 2:** Dados da figura 4 apresentados como média (desvio padrão)

Variáveis	Grupo Controle		Grupo Experimental		Efeito de tempo	Efeito de grupo	Efeito de interação
	Pré	Pós	Pré	Pós			
Fluxo sanguíneo cerebral, mL/min	52,0 (0,2)	54,3 (0,2)	52,1 (0,2)	52,2 (0,3)	<0,001	<0,001	<0,001
Cognitivo, escore	18,8 (0,6)	20,3 (0,7)	19,1 (0,6)	20,2 (0,8)	<0,001	0,488	0,213

Além disso, embora as intervenções tenham o objetivo de melhorar o desfecho principal, é possível que também possam causar efeitos indesejados (reações adversas) em alguns indivíduos. Ainda assim, a comparação das médias, representada em números, não permite a identificação de efeitos indesejáveis após a intervenção. Por exemplo, a figura 5 (painel A) mostra que, após intervenção, houve melhora estatisticamente significativa na função renal − valores pré: 97,1 (2,1) *versus* valores pós: 98,9 (3,7), com p=0,040.

**Figura 5:** Valores pré- e pós-intervenção para função renal (painel A) e dados visuais das respostas individuais. Verde indica melhora, amarelo manutenção, e vermelho piora (painel B)

No entanto, 25% (n=5) dos participantes não apresentaram melhora e 15% (n=3) tiveram reações adversas, conforme mostrado na figura 5 (painel B).

O uso de dados visuais pode favorecer a divulgação de resultados de ECR por meio da comunicação não científica, como televisão, *blogs* e redes sociais − mídias frequentemente usadas para informar o público não acadêmico interessado em pesquisa científica.^(^^4^^)^ Este é um assunto importante, considerando-se o interesse crescente das revistas científicas na divulgação de artigos ao público geral, por meio de mídias sociais (por exemplo: Facebook, Twitter e Instagram).^(^^5^^)^

Nesse sentido, a proposta atual é uma primeira sugestão de como usar dados visuais como ferramenta para melhorar a interpretação dos resultados de estudos clínicos. Pesquisadores e médicos são convidados a usar essa ferramenta em diferentes áreas, para identificar sua viabilidade em diferentes contextos, permitindo que o uso desses dados visuais seja melhorado e refinado.

## DEFINIÇÃO DOS PONTOS DE CORTE

A maioria dos médicos preferem resultados dicotômicos para compreender os resultados dos ECR.^(^^2^^)^ Sabe-se que, para alguns desfechos, esse tipo de resultado pode deixar dúvidas quanto à interpretação dos achados.^(^^3^^)^ O ponto de corte é definido como um ponto principal nos dados visuais. Vários métodos já foram propostos para definir o efeito clinicamente relevante individual das intervenções e, dependendo do método empregado, a interpretação dos dados visuais varia drasticamente.

Seguem os principais métodos que já foram usados para classificar os efeitos das intervenções:

– Delta zero: para alguns desfechos que costumam mudar com o tratamento, se o delta (valores pré e pós) for diferente de zero, isso indica que houve resposta.^(^^6^^)^ Esse método pode facilmente ser contestado, porque não considera variações como a confiabilidade, a variabilidade aleatória e as variações individuais.
– Alterações com base no risco: para algumas variáveis de saúde, estudos longitudinais estabeleceram pontos de corte associados a eventos de saúde. Nesse sentido, alguns estudos usaram esses valores clinicamente relevantes para classificar os desfechos no contexto de ECR. Por exemplo, é possível classificar os participantes como respondedores caso a pressão arterial tenha diminuído para 3mmHg, que é o valor associado a eventos cardiovasculares fatais ou não fatais. Esse critério poderia ser usado para outras variáveis, como rigidez arterial (1m/s),^(^^7^^)^ frequência cardíaca de repouso (75bpm),^(^^8^^)^ dilatação fluxo-mediada (1%)^(^^9^^)^ e biomarcadores,^(^^10^^)^ entre outros.
– Tercis e quartis: os desfechos não têm pontos de corte estabelecidos, e alguns estudos usaram valores arbitrários baseados na mediana, em tercis, quartis ou percentis, o que poderia ser contestado.^(^^11^^,^^12^^)^
– Diferença mínima detectável: define a diferença entre as médias de um tratamento e o controle que deve existir para se concluir que existe um efeito significativo, indo além de qualquer erro de medição com um determinado nível de confiança, geralmente com o nível de confiança de 95%. Para isso, é preciso conhecer o erro da medição da variável sob análise.^(^^13^^)^
